# Beyond Lymphovascular Invasion: Site-Dependent Patterns of Lymphatic, Venous and Perineural Involvement in Laryngo-Hypopharyngeal Carcinoma

**DOI:** 10.3390/medsci14050527

**Published:** 2026-08-28

**Authors:** Roxana-Andreea Popa, Cosmin-Gabriel Popa, Delia Hînganu, Marius Valeriu Hînganu, Cristinel Ionel Stan, Rares-Vasile Tracicaru, Otilia Boișteanu

**Affiliations:** 1 Department of Morpho-Functional Sciences I, Faculty of Medicine, Grigore T. Popa University of Medicine and Pharmacy, 700115 Iasi, Romania; roxana.popa@umfiasi.ro (R.-A.P.); cosmin-gabriel.popa@umfiasi.ro (C.-G.P.); cristinel.stan@umfiasi.ro (C.I.S.); 2 Department of Surgery, Faculty of Medicine, Grigore T. Popa University of Medicine and Pharmacy, 700115 Iasi, Romania; rares-vasile.tracicaru@umfiasi.ro (R.-V.T.); otilia.boisteanu@umfiasi.ro (O.B.)

**Keywords:** laryngeal carcinoma, hypopharyngeal carcinoma, lymphovascular invasion, perineural invasion, vocal fold microvascularization, neurovascular microenvironment, TNM staging, clinico-pathological correlation

## Abstract

Background: Building on our recent characterization of a layer-specific neurovascular microenvironment in the normal human vocal fold, we examined how that unit behaves in laryngo-hypopharyngeal carcinoma, where lymphatic and venous involvement are conventionally reported together. Methods: We retrospectively analyzed 48 laryngo-hypopharyngeal squamous cell carcinomas (2017–2025) with complete TNM vascular reporting, confirmed by CD31 and neuron-specific enolase (NSE) immunohistochemistry. Lymphatic (L) versus venous (V) assignment rested on morphological criteria, since CD31 cannot discriminate lymphatic from blood vessels and D2-40 was unavailable. L, V and perineural (Pn) status were related to pT stage and topography and combined into a composite score (NV = L + V + Pn), reassessed after adjustment for pT. Results: Concordant patterns predominated (L0V0 60.4%, L1V1 27.1%); 12.5% were dissociated. Vascular invasion correlated with pT (ρ = 0.393, *p* = 0.006) and, unadjusted, with Pn, but not after adjustment for pT. NV tracked pT more strongly than any single axis (KR-20 = 0.725; ρ = 0.534, *p* < 0.001). Glottic-only carcinoma showed the lowest involvement on all axes. Conclusions: Reporting L, V and Pn separately—parameters already in routine TNM notation—may capture tumor aggressiveness more completely than LVI as a single entity. This series cannot establish stage-independent co-variation in these axes, validate the L/V distinction molecularly, or, lacking follow-up, demonstrate prognostic value.

## 1. Introduction

The microvascular architecture of the larynx and hypopharynx is regionally specialized to serve the phonatory and deglutitive apparatus. The true vocal cord shows a parallel, tree-like vascular organization, with capillaries concentrated in the superficial lamina propria (Reinke’s space) and no lymphatic drainage at this level [1,2]. This contrasts sharply with the abundant, predominantly lymphatic vascularization of the supraglottis and hypopharynx, organized for rapid drainage toward the cervical lymph node chains [3], while the subglottis occupies an intermediate position, with deep venous networks and inferiorly oriented lymphatics [2,3]. Crucially, this regional heterogeneity is accompanied by an integrated neurovascular organization in which vessels and nerves are co-distributed and regionally regulated [4,5]—implying that the response of these structures to pathological stress, including the tumor microenvironment, may itself be regionally differentiated.

In oncological practice, blood and lymphatic vessels are analyzed mainly as routes of tumor dissemination. Lymphovascular invasion (LVI) and perineural invasion (Pn) are established histopathological prognosticators in upper aerodigestive tract carcinoma and influence adjuvant decisions [6,7,8], yet this framing casts vessels in a passive role rather than as active participants with their own regionally differentiated behavior.

Two lines of evidence indicate that this framing deserves revision. In esophageal carcinoma, dual immunohistochemistry (CD31 for blood vessels and D2-40/podoplanin for lymphatic endothelium) has shown that lymphatic and venous invasion can be distinct and may carry different prognostic weight, challenging the treatment of LVI as a single category [9]. In parallel, recent syntheses of laryngeal squamous cell carcinoma emphasize that LVI continues to be reported as an aggregated entity, despite the distinct architecture of lymphatic and venous vessels [10]. Importantly, the UICC/AJCC TNM system already permits separate reporting of the two components through the L (lymphatic) and V (venous) notations, offering an opportunity to analyze differentiated patterns of vascular involvement without additional immunohistochemistry.

These two compartments differ structurally in ways that plausibly condition their response to the tumor: lymphatic vessels have a discontinuous basal lamina, lack circumferential smooth muscle, and are intrinsically permeable, whereas small venous vessels present a more stable, continuous wall [11,12,13]. Such differences make a differentiated behavior of the two compartments a testable clinical hypothesis rather than an assumption.

The neural structures adjacent to these vessels are frequently involved through perineural invasion, an independent predictor of local recurrence and reduced survival in laryngeal carcinoma [10]. Although Pn has traditionally been regarded as biologically separate from vascular invasion, the tightly integrated neurovascular anatomy of the larynx [4,14] suggests that vessels and nerves form a functional unit that may share a co-localized vulnerability to tumor stress.

Despite this rationale, a systematic analysis of vascular invasion disaggregated by the component (L vs. V), correlated with perineural invasion and with precise tumor topography (glottic vs. supraglottic vs. transglottic vs. hypopharyngeal), remains poorly represented. Most clinico-pathological studies report LVI as a unitary variable and use coarse topographical categories that do not separate glottic-only carcinoma from multi-level disease—a methodological gap that limits the integration of clinico-pathological observation with the anatomical basis of laryngeal microvascularization synthesized in our previous review [15,16].

Recently, our group demonstrated that the normal human vocal fold contains a highly organized layer-specific neurovascular microenvironment, characterized by the parallel spatial distribution of microvascular, neural and extracellular matrix components. This observation suggested that vessels and nerves do not represent independent anatomical entities but rather components of an integrated neurovascular functional unit. The present study extends this concept into pathology by investigating how this neurovascular unit behaves in laryngo-hypopharyngeal carcinoma [17].

**Figure 1:**
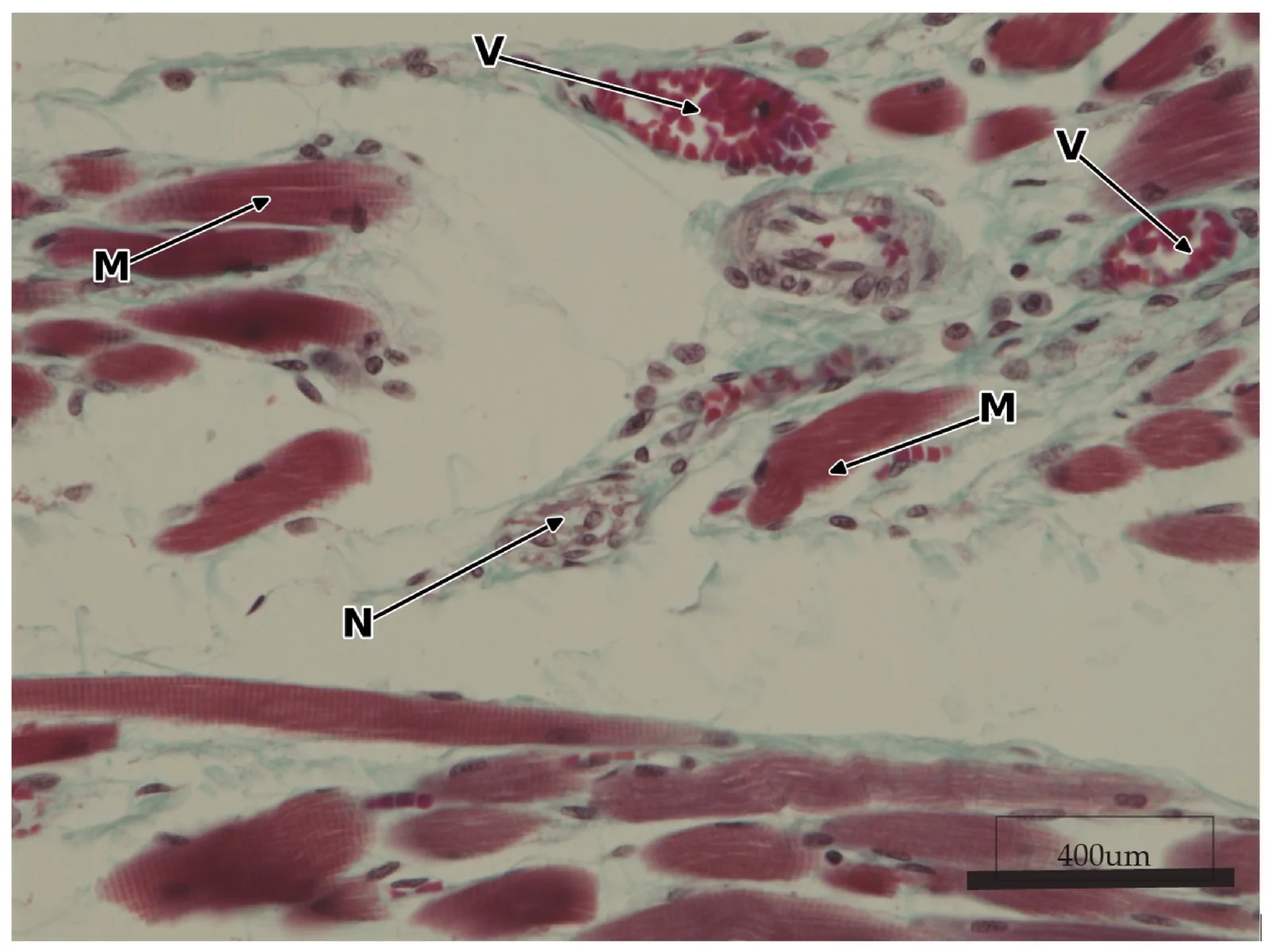
Histoarchitectural organization of the laryngeal intramuscular neurovascular unit. Goldner trichrome section of the human myolaryngovocalis muscle showing the close spatial relationship between an intramuscular blood vessel (V), a peripheral nerve bundle (N), and adjacent skeletal muscle fascicles (M). The neurovascular structures course together within the connective tissue septa that separate adjacent muscle fascicles. Arrows indicate an intramuscular blood vessel (V), a peripheral nerve bundle (N), and skeletal muscle fascicles (M). Original magnification ×100; the scale bar is shown in the figure. Archival preparation of a normal human larynx (Anatomical Institute, Christian Albrechts University, Kiel, Germany). This specimen is not part of the tumor cohort analyzed in the present study and is shown solely to illustrate the normal anatomical background of the neurovascular unit.

Representative Goldner trichrome sections illustrating the close spatial relationship between intramuscular blood vessels (V), peripheral nerve bundles (N), and skeletal muscle fascicles (M). The neurovascular elements course together within the connective tissue septa separating adjacent muscle fascicles, forming reproducible intramuscular neurovascular complexes. These normal anatomical relationships provide the structural background for the integrated neurovascular concept investigated in the present study (Figure 1).

The present study investigates how this anatomical-functional unit behaves in carcinoma. To this end, we analyze a retrospective series of 48 laryngo-hypopharyngeal squamous cell carcinomas, selected on the basis of complete TNM reporting of vascular status, and examine the patterns of L + V invasion in relation to perineural invasion (Pn), pT stage, and precise tumor topography. In doing so, we position the vessels—not the tumor—as the principal actor: their behavior in the tumor context reflects their own regional architecture, functional state, and relationship with neighboring neural structures, providing an interpretive framework that connects the recently characterized laryngeal neurovascular anatomy [15,17] with routine clinico-pathological observation.

## 2. Materials and Methods

The present study is a retrospective, observational, single-center analysis aimed at characterizing the patterns of vascular invasion in laryngo-hypopharyngeal squamous cell carcinoma. The data were obtained from the electronic archive of the Department of Pathology of the “Sf. Spiridon” Emergency Clinical Hospital, Iași, Romania, covering a period of nine consecutive years (January 2017–December 2025).

The initial cohort included 247 consecutive cases with laryngo-pharyngeal pathology diagnosed in this period. For the final selection, the following inclusion criteria were applied: (i) histopathological diagnosis of squamous cell carcinoma (WHO code 8070/3 or related variants: 8071/3, 8072/3, 8083/3); (ii) primary laryngeal or hypopharyngeal localization confirmed macroscopically and microscopically; (iii) pathology reporting containing TNM notation with at least one of the L (lymphatic) or V (venous) components, according to the UICC/AJCC TNM system.

Exclusion criteria: (i) carcinomas with primary extralaryngeal origin (facial skin, oral cavity, major salivary glands, paranasal sinuses, nasopharynx), identified by ICD-10 codes C00–C09, C11, C14, C30, C44; (ii) cases with secondary laryngeal invasion from an extralaryngeal–extrahypopharyngeal primary tumor; (iii) cases without detailed histopathological description or without standardized TNM notation.

The final study group included 48 cases of laryngo-hypopharyngeal squamous cell carcinoma with complete TNM reporting of vascular status (L and/or V), representing the pivot criterion for inclusion in the study.

### 2.1. Ethical Approval

The study was conducted in accordance with the Declaration of Helsinki. Ethical approval was obtained from the Ethics Committee of the “Sf. Spiridon” Emergency Clinical Hospital, Iași, and from the Ethics Committee of the “Grigore T. Popa” University of Medicine and Pharmacy, Iași (protocol code 304; approval date: 16 May 2023). Given the retrospective nature of the study and the exclusive use of anonymized data from the hospital archive, individual informed consent was waived.

### 2.2. Extraction of Demographic and Clinico-Pathological Data

For each included case, the following variables were collected: age at the time of intervention, sex, area of residence (rural/urban), year of diagnosis, type of surgical intervention (biopsy, cordectomy, hemilaryngectomy, total laryngectomy, pharyngo-laryngectomy), primary and secondary ICD-10 coded diagnoses, morphological diagnosis (WHO code), macroscopic and microscopic description of the specimen, resection margin status (R0/R1/R2), and TNM parameters.

The TNM parameters systematically extracted included: pathological tumor stage (pT, according to the UICC/AJCC 8th edition classification [18]), nodal stage (pN), histological grade of differentiation (G), lymphatic invasion (L), venous invasion (V), perineural invasion (Pn), and surgical margins (R). The “y” prefix (neoadjuvant therapy) was recorded separately when present. For standardization of statistical analysis, pT stage was grouped into four categories (T1, T2, T3, T4), reuniting T1a and T1b subcategories under T1, and T4a and T4b under T4.

### 2.3. Determination of Tumor Topography

**Figure 2:**
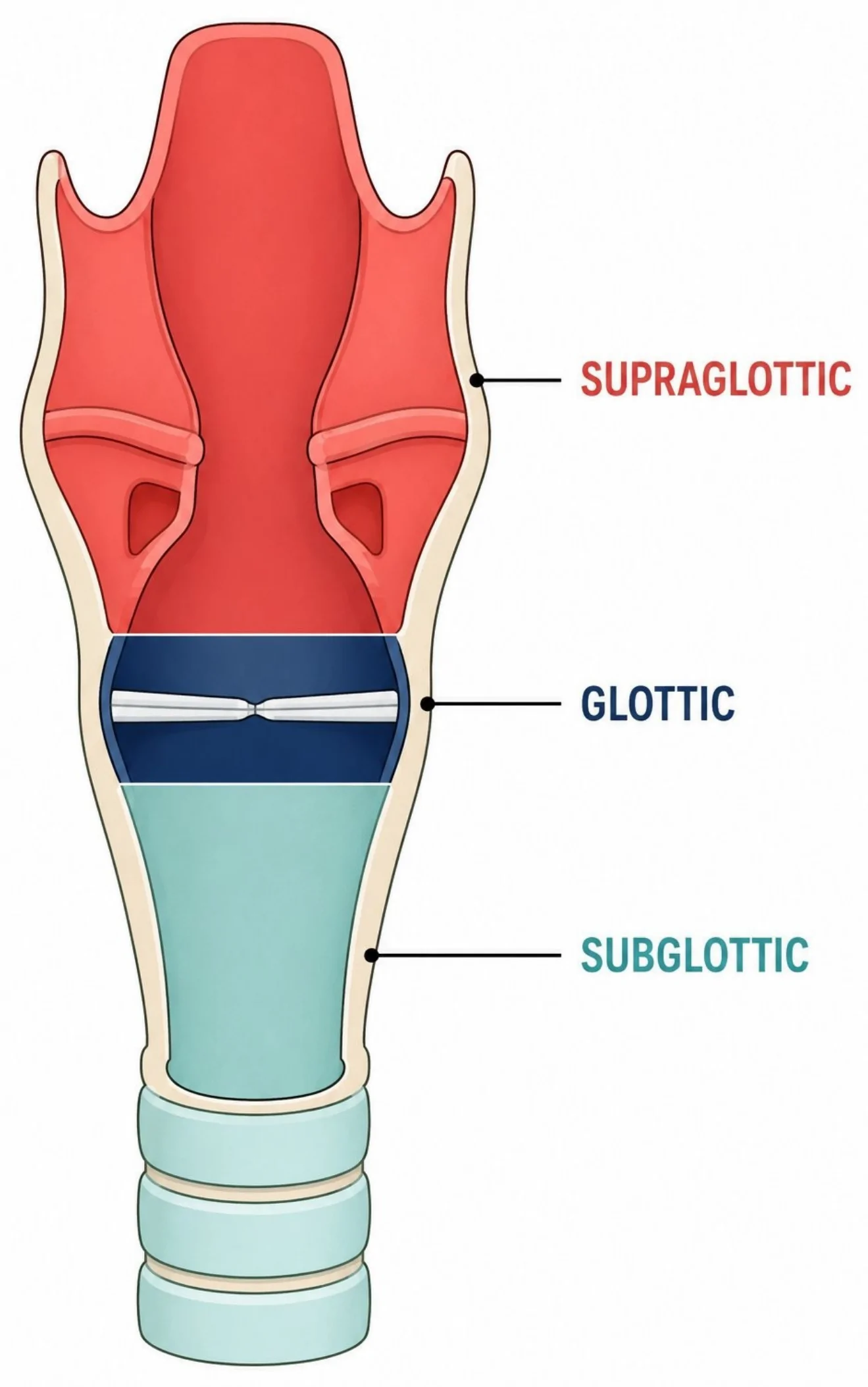
Schematic representation of the three anatomical levels of the larynx used in AJCC tumor staging: supraglottic, glottic, and subglottic. The schematic illustrates the spatial relationship between the supraglottic region (containing the epiglottis, false vocal cords, and laryngeal ventricles), the narrow glottic region (containing the true vocal cords), and the subglottic region continuous with the cervical trachea. Original schematic drawing produced by the authors.

The three anatomical levels of the larynx used for topographic classification and AJCC staging are represented schematically in Figure 2. Tumor topography was established through systematic analysis of relevant textual fields: macroscopic description, microscopic description, specimen description, reported surgical intervention, primary and secondary diagnoses, and the epicrisis. This cumulative approach was necessary because the precise anatomical localization is frequently specified in different fields of the pathology report.

Identification of anatomical areas followed a standardized clinico-anatomical nomenclature. Glottic areas (glottic level): true vocal cord, anterior commissure, posterior commissure, paraglottic space. The mention of “vocal cord” or “vocal cords” without the qualifier “false” was interpreted as referring to the true vocal cord, according to standard clinico-pathological practice. Supraglottic areas (supraglottic level): false vocal cord (ventricular band), Morgagni’s ventricle, epiglottis, vallecula, aryepiglottic fold, arytenoid, laryngeal vestibule. Subglottic areas (subglottic level): the region located inferior to the true vocal cord, including references to cricoid cartilage when tumor invasion was specified. Hypopharyngeal areas: pyriform sinus, retrocricoid region, posterior hypopharyngeal wall.

Based on the identified areas, each case was classified into a unique topographic category reflecting the primary level and tumor extension: glottic only (limited to the glottic level); supraglottic only (limited to the supraglottic level); subglottic only (limited to the subglottic level); glottic + subglottic (combined glottic and subglottic involvement, without supraglottic); supraglottic + glottic (combined supraglottic and glottic involvement, without subglottic); transglottic (involvement of all three laryngeal levels: glottic + supraglottic + subglottic); hypopharynx only (limited to the pyriform sinus or other hypopharyngeal structures); hypopharynx + larynx (hypopharyngeal origin or extension with concomitant laryngeal invasion).

For inferential statistical analysis, given the small-volume subgroups, topographies were grouped into four main categories: glottic only, multi-level laryngeal (transglottic + glottic + subglottic + supraglottic + subglottic), supraglottic (±extension) (supraglottic only + supraglottic + glottic), and hypopharynx (±larynx) (hypopharynx only + hypopharynx + larynx).

### 2.4. Extraction of Vascular (L, V) and Perineural (Pn) Invasion Status

The pattern of vascular invasion was extracted based on the standardized TNM notation present in the pathology report, following the UICC/AJCC criteria. Three reporting formats were identified in the analyzed archive:

(1) Complete explicit format (L_x_V_x_, e.g., “L0V0”, “L1V1”, “L1V0”, “L0V1”): lymphatic and venous components reported separately, with potentially different values. (2) Abbreviated format (LV_x_, e.g., “LV0”, “LV1”): notation used by the pathologist when both components (lymphatic and venous) presented the same value. “LV0” was interpreted as L0V0 (absence of invasion on both components), and “LV1” as L1V1 (presence of invasion on both components). This convention was validated through discussion with the pathologists who issued the reports in the analyzed archive. (3) Separate format (L and V mentioned distinctly in the text, e.g., “L0 V0”). This classification allowed the complete recovery of all cases with vascular TNM reporting, regardless of the syntactic variant used in the report.

Based on the L and V status, each case was assigned to one of four vascular patterns: L0V0—absence of vascular invasion; L1V1—concurrent lymphatic and venous invasion (concordant positive pattern); L1V0—isolated lymphatic invasion (dissociated, predominantly lymphatic pattern); L0V1—isolated venous invasion (dissociated, predominantly venous pattern). The perineural invasion status (Pn0/Pn1) was extracted independently from the same TNM notation, when available.

### 2.5. Immunohistochemical Confirmation of Vascular and Perineural Invasion

To strengthen the validity of the histopathological reporting and to ensure consistent identification of vascular and perineural invasion across the study group, immunohistochemical confirmation was performed on all 48 cases using CD31 for vascular endothelium and NSE (neuron-specific enolase) for neural structures.

Endothelial cells were identified using CD31 (platelet endothelial cell adhesion molecule-1, PECAM-1). A mouse monoclonal anti-CD31 antibody (clone JC/70A, Abcam, Cambridge, UK) was applied at a dilution of 1:500. Antigen retrieval was performed using citrate buffer (pH 6.0). Sections were incubated with the primary antibody overnight at 4–6 °C. Immunodetection was performed using a polymer-based horseradish peroxidase (HRP)/DAB detection system (Mouse and Rabbit Specific HRP/DAB Detection Kit, ab64264, Abcam, Cambridge, UK), compatible with formalin-fixed, paraffin-embedded tissues. Visualization was achieved using 3,3′-diaminobenzidine (DAB) as chromogen. Sections were counterstained with hematoxylin, dehydrated, cleared, and mounted for microscopic evaluation. Positive controls consisted of vessel-rich tissue sections with known CD31 expression. Negative controls were performed by omission of the primary antibody.

To evaluate the neural component of the tumor microenvironment, immunohistochemical staining for neuron-specific enolase (NSE) was performed on representative sections from all 48 cases. Antigen retrieval was carried out using an epitope retrieval solution at pH 9, according to the manufacturer’s recommendations for the antibody system used. Sections were then incubated overnight at 4–6 °C with a ready-to-use primary anti-NSE antibody (Leica Biosystems; clone 22C9; mouse monoclonal). Immunodetection was achieved using a polymer-based HRP/DAB detection system, with diaminobenzidine as chromogen. Sections were counterstained with hematoxylin. Positive controls consisted of tissue sections with known NSE expression; negative controls were obtained by omission of the primary antibody. For both markers, the polymer-based HRP reagent is supplied ready-to-use, so no separate secondary-antibody dilution was prepared, and detection was carried out according to the manufacturer’s protocol. NSE-positive structures were identified as neural elements, including nerve fibers and neural profiles within the tumor stroma. The assessment of NSE expression was performed using a quantitative morphometric approach, complemented by qualitative evaluation of distribution patterns.

All slides were independently evaluated by two pathologists blinded to the original TNM reporting. CD31 positivity confirmed the presence of vascular structures invaded by tumor cells (lymphovascular invasion), while NSE positivity confirmed the neural identity of structures surrounded by tumor cells in cases reported as perineural invasion. All 48 cases were assessed, with full concordance between the original TNM-based reporting and the immunohistochemical confirmation—no case required reclassification.

### 2.6. Goldner Trichrome Reference Sections

Representative Goldner trichrome sections of the normal human vocal fold were included exclusively for anatomical illustration of the normal neurovascular organization. These preparations were not part of the retrospective tumor cohort and were not included in the quantitative or statistical analyses performed in the present study.

To provide the normal histoarchitectural background of the laryngeal neurovascular unit, archival Goldner trichrome sections from normal human larynges were included for anatomical illustration. The material originated from five adult human larynges (2 males, 3 females; age range 67–81 years) donated by written testamentary disposition to the Anatomical Institute of Christian-Albrechts-University (CAU), Kiel, Germany, in accordance with German legislation governing body donation. The larynges had been fixed in 4% formalin, embedded in paraffin, sectioned in the sagittal and coronal planes (7 μm), and stained with Goldner trichrome using standard histological protocols. These preparations were used exclusively to illustrate the normal spatial relationship between intramuscular vessels, peripheral nerves, and the myolaryngovocalis muscle and were not included in the quantitative or statistical analyses of the present study.

### 2.7. Statistical Analysis

Statistical analysis was performed using Python 3.12 (pandas, scipy, statsmodels libraries). Descriptive data are presented as means and standard deviations (quantitative variables) or as absolute and relative frequencies (categorical variables).

For evaluating associations between categorical variables, the following tests were used: Fisher’s exact test for 2 × 2 tables with reduced expected frequencies (<5); the Fisher–Freeman–Halton exact test for r × 2 contingency tables with expected frequencies below; McNemar’s test for evaluating the symmetry of discordance between lymphatic (L) and venous (V) invasion in the same patient. For evaluating correlations between ordinal variables (pT) and binary variables (L, V, Pn, overall LVI), the Spearman rank correlation coefficient (ρ) was used, with two-tailed significance testing.

The statistical significance threshold was set at *p* < 0.05. Results with *p*-values between 0.05 and 0.10 were interpreted as statistical trends and explicitly mentioned as such, without being presented as significant. Given the exploratory nature of the study and the relatively reduced dimensions of some topographical subgroups, no corrections for multiple testing were performed, and the interpretation of results respected the principle of prudence in formulating conclusions, in accordance with recommendations for retrospective observational studies with medium-sized cohorts.

To evaluate the lymphatic (L), venous (V), and perineural (Pn) axes as indicators of a single integrated compartment, a composite neurovascular-unit score (NV) was defined as the sum of the three binary components (NV = L + V + Pn; range 0–3), computed for the 47 cases with reported Pn. Internal consistency of the three-item construct was assessed using the Kuder–Richardson 20 coefficient (KR-20, equivalent to Cronbach’s α for dichotomous items). Differences in the NV score across grouped topographies were tested with the Kruskal–Wallis test; the a priori hypothesis that glottic-only carcinoma exhibits the lowest neurovascular-unit involvement was tested directionally with the Mann–Whitney U test (glottic-only vs. all other topographies), with the two-sided value also reported.

Because perineural and vascular invasion both correlate with tumor stage, the association between them was additionally examined after adjustment for pT. Partial Spearman correlations were computed on rank-transformed variables with pT as a covariate, and a multivariable logistic regression model was fitted with Pn1 as the dependent variable and the number of involved vascular axes (0–2) and pT stage as predictors. A Cochran–Mantel–Haenszel test stratified by pT was used as a non-parametric confirmation. Given the limited number of events (13 Pn1 cases), these adjusted analyses are considered exploratory and are reported with 95% confidence intervals rather than interpreted through significance thresholds alone.

## 3. Results

### 3.1. Demographic and Clinico-Pathological Characteristics of the Study Group

The final study group included 48 patients with laryngo-hypopharyngeal squamous cell carcinoma and complete TNM reporting of vascular status. The demographic and clinico-pathological characteristics are summarized in Table 1.

The mean age at the time of intervention was 61.3 ± 7.6 years (median 62, range 37–78). The sex distribution evidenced a marked male predominance, with 46 male patients (95.8%) and only 2 female patients (4.2%), reflecting the classical epidemiological profile of laryngeal carcinoma. The distribution by residence area was relatively balanced, with a slight rural predominance (27 cases, 56.2%) versus urban (21 cases, 43.8%).

Cases were distributed relatively uniformly over the study period (2017–2024), with a minimum in 2020 (*n* = 2, probably attributable to the COVID-19 pandemic) and maxima of 8 cases in 2022 and 2023.

The pT stage distribution revealed a significant representation of advanced stages: T1 (*n* = 16, 33.3%), T2 (*n* = 4, 8.3%), T3 (*n* = 14, 29.2%), and T4 (*n* = 14, 29.2%). The combined T3–T4 cases represented 58.4% of the study group. The histological grade was predominantly moderately differentiated (G2, *n* = 25, 52.1%), followed by well-differentiated (G1, *n* = 11, 22.9%); the grade was not reported in 10 cases (20.8%).

### 3.2. Tumor Topographic Distribution

The topographic analysis of tumors revealed a heterogeneous distribution, with a net predominance of multi-regional extensive localizations (Table 2). The most frequent category was represented by tumors with hypopharyngeal involvement and laryngeal extension (hypopharynx + larynx), totaling 26 cases (54.2%). Glottic-only carcinoma, limited to the true vocal cord, was identified in 7 patients (14.6%), a percentage comparable to that of transglottic tumors (*n* = 7, 14.6%). The supraglottic + glottic and glottic + subglottic localizations represented 10.4% and 6.2% of the study group, respectively.

### 3.3. TNM Reporting Format and Vascular Status

The overall rate of lymphatic invasion (L1), as recorded in the TNM notation, in the study group was 35.4% (17/48), and that of venous invasion (V1) was 31.2% (15/48). Perineural status (Pn) was reported in 47 cases (97.9%), with a Pn1 rate of 27.7% (13/47).

### 3.4. Immunohistochemical Confirmation of Vascular and Perineural Invasion

Immunohistochemical staining with CD31 (endothelial marker) and NSE (neural marker) was performed on all 48 cases of the study group. CD31 positivity confirmed the endothelial nature of vascular structures invaded by tumor cells in all cases reported as L1 or V1 in the original TNM notation. NSE positivity confirmed the neural identity of structures invaded by tumor cells in all cases reported as Pn1. The concordance between the original histopathological reporting and the immunohistochemical confirmation was complete (100%), with no case requiring reclassification.

**Figure 3:**
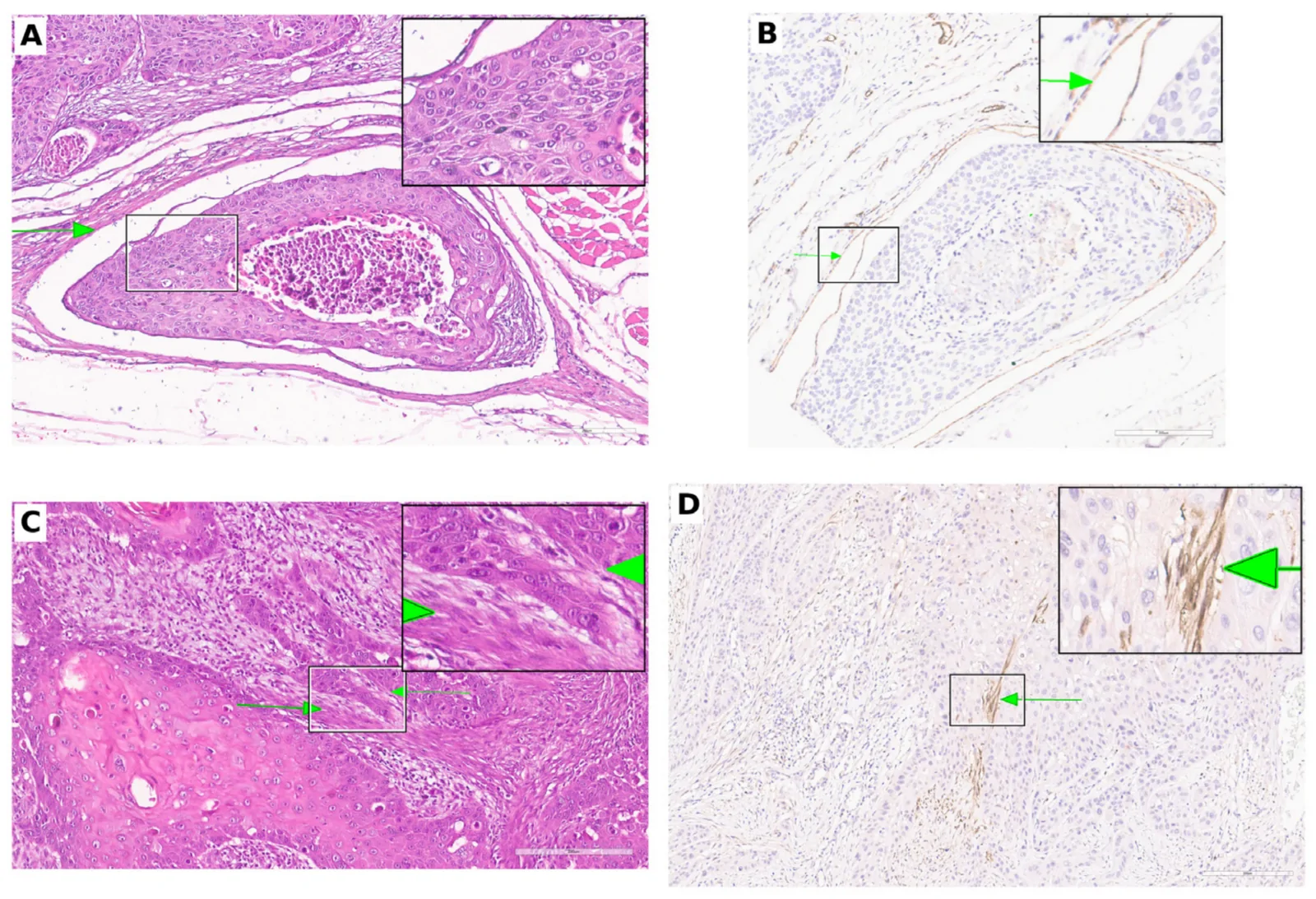
Paired histological and immunohistochemical validation of vascular and perineural invasion. Upper row—vascular invasion: (**A**) Hematoxylin–eosin: tumor embolus within a venous vessel (arrow). (**B**) CD31 immunohistochemistry: membranous endothelial positivity of the same vessel wall (arrow), confirming the vascular nature of the invaded structure. Lower row—perineural invasion: (**C**) Hematoxylin–eosin: tumor cells (arrows) surrounding nerve bundles at the invasion front. (**D**) NSE immunohistochemistry: cytoplasmic positivity confirming the neural identity of the invaded structures (arrows). In each panel, the boxed area is shown enlarged in the corresponding inset (approximately 2–3× the panel magnification); insets are digital magnifications of the same image, without any adjustment of brightness, contrast or color. Scale bars are shown in each panel and apply to the main panels, not to the insets. CD31 = platelet endothelial cell adhesion molecule-1; NSE = neuron-specific enolase. Immunohistochemistry was performed to verify the endothelial and neural identity of the structures already identified on hematoxylin–eosin, not to re-screen the slides for additional foci.

Representative paired histological and immunohistochemical findings are illustrated in Figure 3. The upper row (Panels A and B) shows the validation of vascular invasion: Panel A displays a large tumor embolus within a venous vessel on hematoxylin–eosin staining, while Panel B confirms the endothelial identity of the vessel wall through CD31 immunohistochemistry, demonstrating the vascular nature of the structure invaded by tumor cells. The lower row (Panels C and D) shows the validation of perineural invasion: Panel C displays perineural invasion at the tumor front on hematoxylin–eosin staining, with tumor cells surrounding nerve bundles, while Panel D confirms the neural identity of these structures through NSE immunohistochemistry. The paired HE/IHC approach provides direct molecular validation of both vascular and perineural invasion patterns reported in the TNM notation.

### 3.5. Vascular Invasion Patterns: Concordance and Dissociation Between Lymphatic and Venous Components

The four possible vascular invasion patterns, as recorded in the TNM notation, presented the following frequencies: L0V0 (absence of vascular invasion)—29 cases (60.4%); L1V1 (concurrent lymphatic and venous invasion)—13 cases (27.1%); L1V0 (isolated lymphatic invasion)—4 cases (8.3%); L0V1 (isolated venous invasion)—2 cases (4.2%). Their distribution across grouped tumor topographies is analyzed in Section 3.8.

The two concordant patterns (L0V0 and L1V1) accounted for 42 of 48 cases (87.5%), confirming the dominant tendency toward parallel involvement of the two vascular compartments. However, 6 cases (12.5%) presented a dissociated pattern, in which the invasion affected only one of the two vascular components. The distribution of discordance was asymmetric: isolated lymphatic invasion (L1V0) was more frequent than isolated venous invasion (L0V1), in a 2:1 ratio (4 vs. 2 cases).

McNemar’s test for the symmetry of discordance between the lymphatic and venous components did not reveal a statistically significant asymmetry (χ^2^ = 0.167, *p* = 0.683), a result reflecting the reduced size of the discordant subgroup (*n* = 6). The overall correlation between L and V status was strong (Spearman ρ = 0.722, *p* = 2.2 × 10^−8^), confirming that, although the two compartments can be affected dissociatively, there is a robust population-level association.

### 3.6. Association of Vascular Pattern with pT Tumor Stage

**Figure 4:**
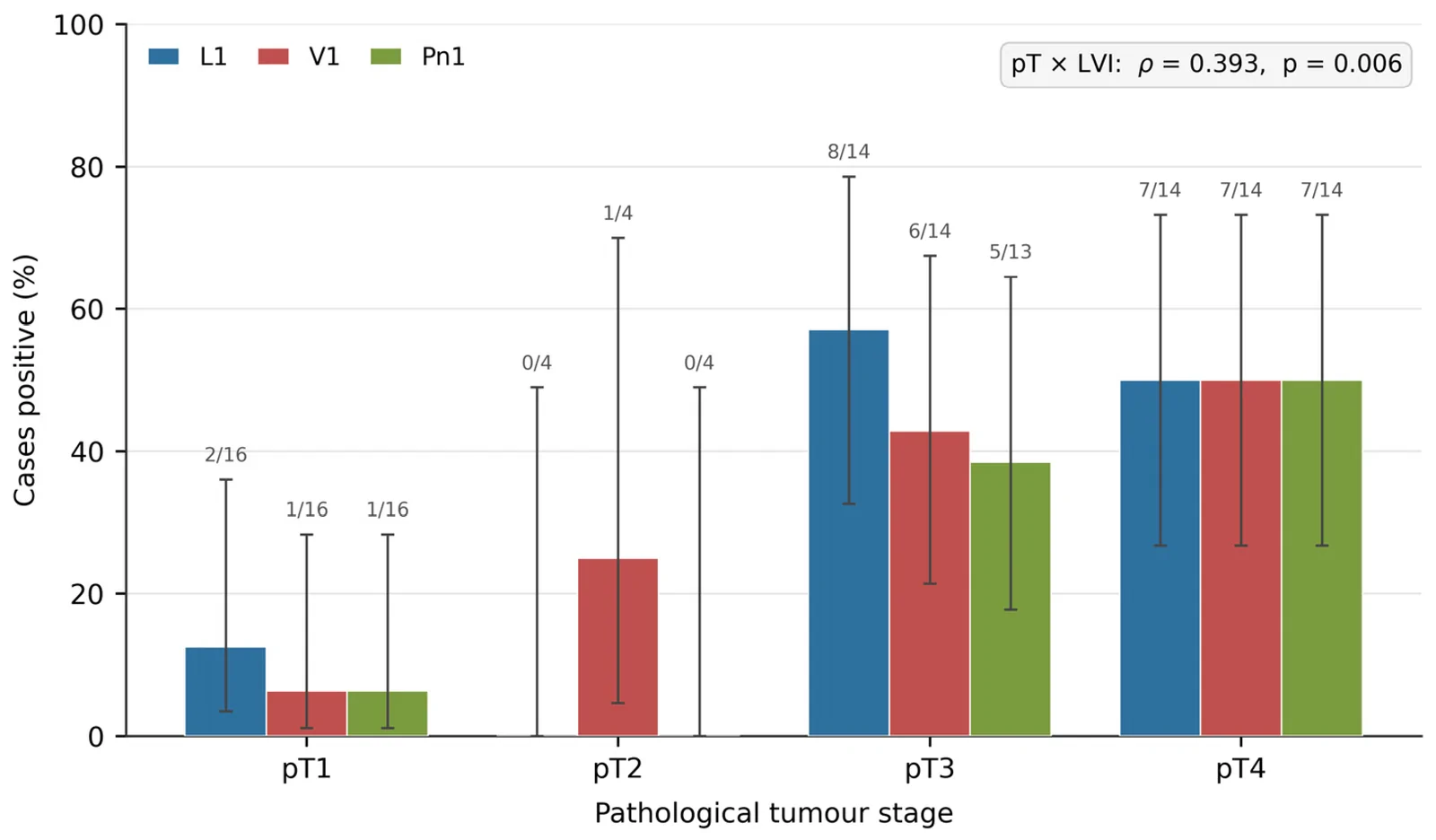
Rates of lymphatic (L1), venous (V1), and perineural (Pn1) invasion by pT tumor stage. Each series shows the proportion of cases positive on the respective axis within each pT stratum (pT1, *n* = 16; pT2, *n* = 4; pT3, *n* = 14; pT4, *n* = 14). Error bars are Wilson 95% confidence intervals for each proportion; absolute case counts (positive/total) are given above each bar. All three axes increase sharply between pT2 and pT3, the transition at which laryngeal anatomical barriers are crossed. The inset reports the Spearman correlation between pT stage and overall vascular invasion (LVI = any L1 or V1): ρ = 0.393, *p* = 0.006. Perineural status was reported in 47 of 48 cases; the single case without Pn reporting belonged to the pT3 stratum (Pn denominator = 13). Percentages for the pT2 stratum (*n* = 4) should be interpreted with caution.

The overall vascular invasion rate (LVI, defined as the presence of L1 or V1) presented a clear ascending gradient with pT stage: T1—12.5% (2/16); T2—25.0% (1/4); T3—57.1% (8/14); T4—57.1% (8/14). The rates of the three individual axes across pT strata are shown in Figure 4.

The Spearman correlation coefficient confirmed a statistically significant association between pT and LVI: ρ = 0.393, *p* = 0.006. The association was maintained when analyzing the components separately: pT–L1 (ρ = 0.365, *p* = 0.011) and pT–V1 (ρ = 0.390, *p* = 0.006) were both significant, with similar magnitudes.

The distribution of specific patterns by pT stage revealed that the concordant positive pattern (L1V1) was rare in early stages (1/16 in T1, 0/4 in T2) and frequent in advanced stages (6/14 in T3, 6/14 in T4). The dissociated patterns (L1V0 and L0V1) were heterogeneously distributed across all stages, without an evident concentration in a specific pT category.

### 3.7. Co-Variation in Perineural Invasion with Vascular Pattern

The analysis of the correlation between Pn status and vascular pattern (*n* = 47 cases with reported Pn) revealed significant associations with both the lymphatic and the venous components of vascular invasion: Spearman Pn–L1: ρ = 0.359, *p* = 0.013; Spearman Pn–V1: ρ = 0.325, *p* = 0.026.

**Figure 5:**
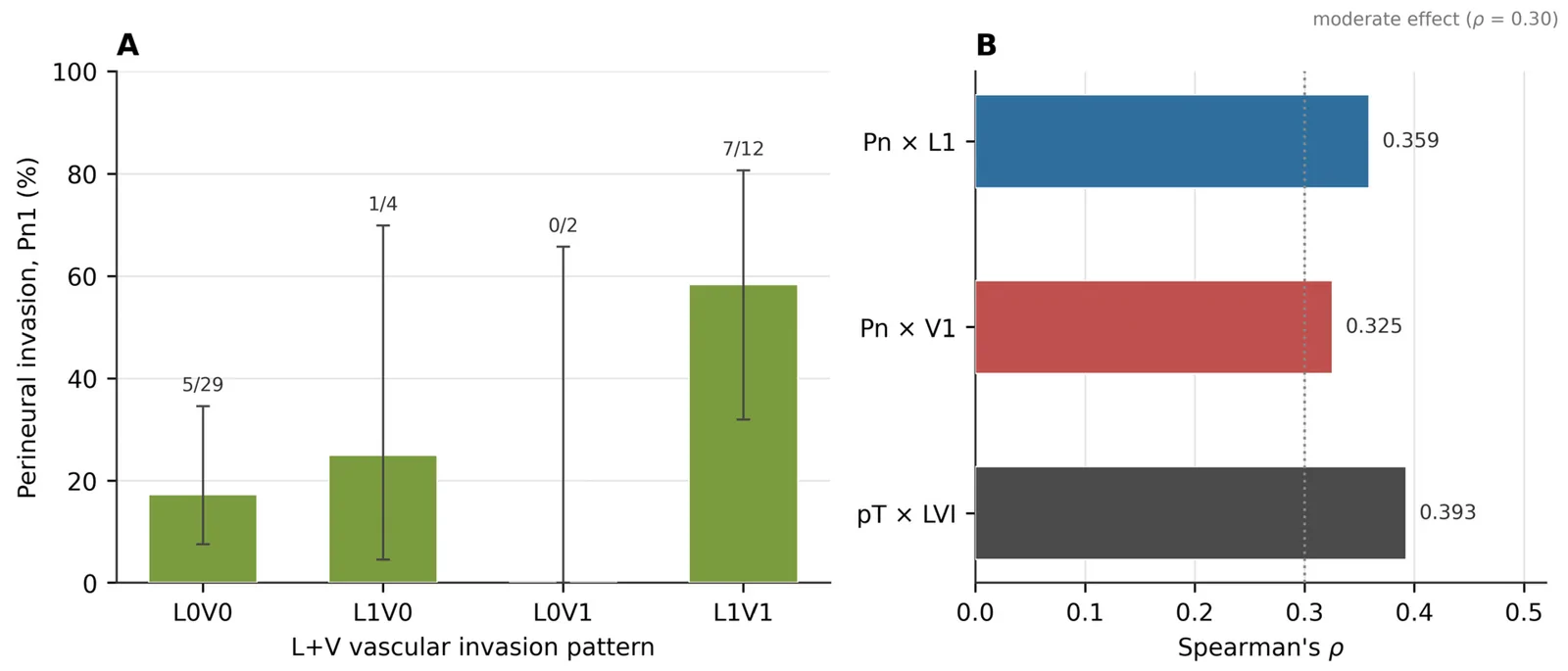
Co-variation between perineural invasion (Pn) and the L + V vascular invasion pattern. (**A**) Proportion of Pn1 and Pn0 cases within each vascular pattern. (**B**) Spearman correlation coefficients for the pT–LVI, Pn–V, and Pn–L associations; the dotted line marks the conventional threshold for a moderate effect size (ρ = 0.30). In panel A, the rate of perineural invasion rises from 17.2% in the L0V0 pattern to 58.3% in the L1V1 pattern, with intermediate values in the dissociated patterns. Error bars are Wilson 95% confidence intervals; absolute case counts (positive/total) are given above each bar. Group sizes refer to the 47 cases with reported Pn status; the single case without Pn reporting had an L1V1 pattern, which is why the L1V1 group comprises 12 rather than 13 cases in this analysis. LVI = overall vascular invasion (any L1 or V1).

The distribution of perineural invasion across the four vascular patterns (Figure 5A) showed that Pn1 was present in 7 of 12 cases with the L1V1 pattern (58.3%), compared to only 5 of 29 cases with the L0V0 pattern (17.2%). The dissociated patterns presented intermediate values: 1/4 (25.0%) for L1V0 and 0/2 (0.0%) for L0V1.

These unadjusted results suggest a co-variation between perineural and vascular invasion, more pronounced with the lymphatic than with the venous component. However, since all three axes correlate independently with pT stage, this association could be confounded by tumor stage. After adjustment for pT, the correlations were attenuated and no longer reached significance (partial Spearman Pn–L1: ρ = 0.242, 95% CI −0.052 to 0.498, *p* = 0.105; Pn–V1: ρ = 0.191, 95% CI −0.105 to 0.456, *p* = 0.203). In the multivariable logistic model, pT stage remained an independent predictor of perineural invasion (OR = 2.27 per stage increment, 95% CI 1.06–4.87, *p* = 0.035), whereas the number of involved vascular axes did not (OR = 1.83, 95% CI 0.81–4.16, *p* = 0.148), compared with OR = 2.54 (*p* = 0.016) in the unadjusted model. A Cochran–Mantel–Haenszel analysis stratified by pT yielded concordant estimates (Pn × L1: OR_MH_ = 2.34, 95% CI 0.57–9.56; Pn × V1: OR_MH_ = 2.49, 95% CI 0.54–11.58).

The direction of the association was therefore preserved across all three analytical approaches, but its magnitude and precision were insufficient to establish independence from tumor stage in a series of this size (13 perineural events; 6.5 events per variable). The co-variation between the neural and vascular axes should accordingly be regarded as a hypothesis supported by the descriptive data rather than as a stage-independent effect demonstrated by this cohort.

### 3.8. Differential Vascular Patterns by Tumor Topography

**Figure 6:**
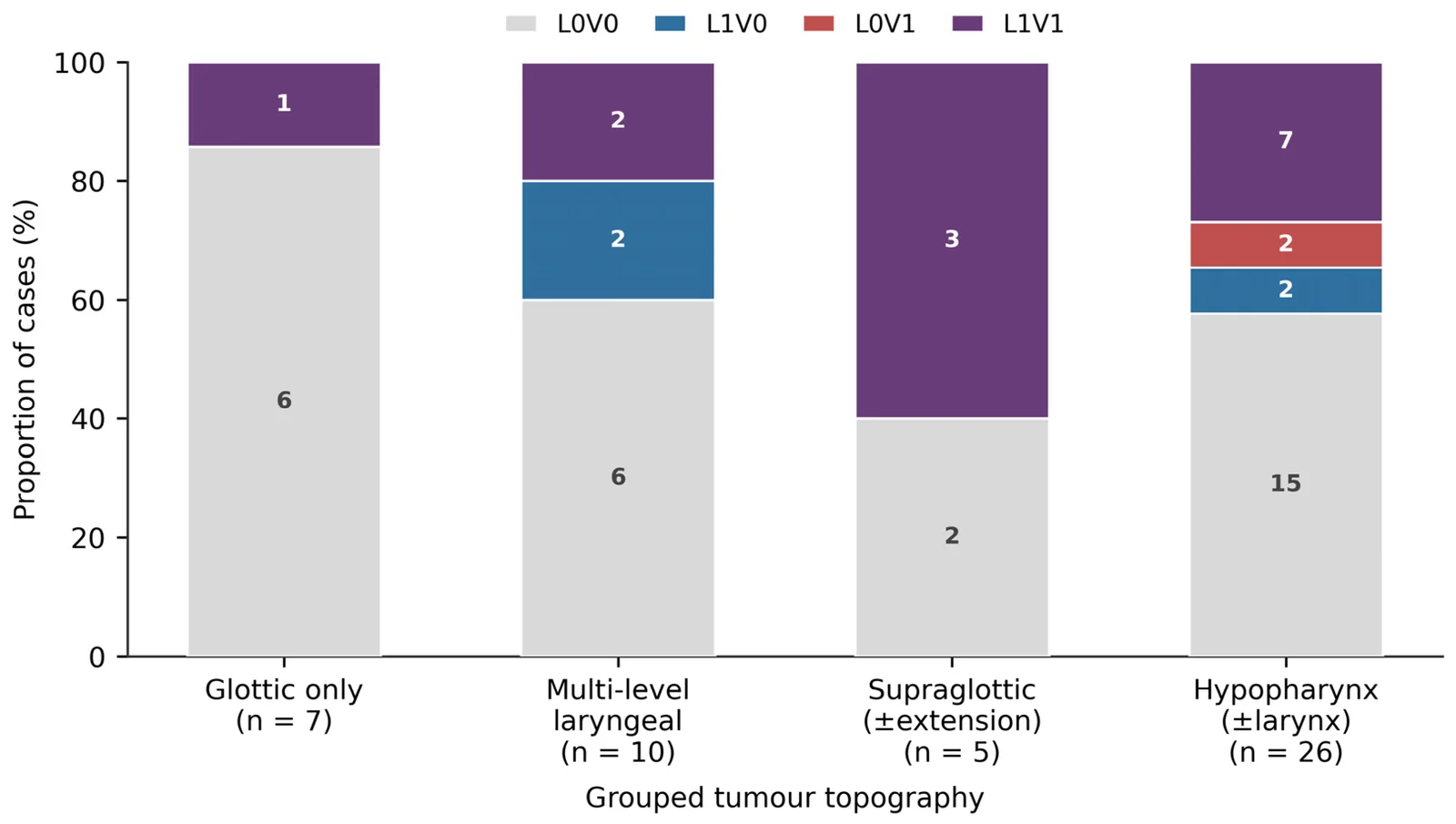
Distribution of L + V vascular invasion patterns across the four grouped tumor topographies. Each bar represents the proportion of vascular invasion patterns (L0V0, L1V0, L0V1, L1V1) within each topographic group. Glottic-only carcinoma is characterized by a strong predominance of the L0V0 pattern (85.7%), whereas supraglottic carcinomas display the highest proportion of the concordant positive pattern L1V1 (60.0%). Exact case counts are given in Table 3. Given the small size of the glottic (*n* = 7) and supraglottic (*n* = 5) subgroups, proportions should be interpreted as descriptive.

The analysis of the vascular pattern distribution by grouped tumor topography revealed a clear gradient of neurovascular involvement across the four anatomical regions (Table 3, Figure 6).

Glottic-only carcinoma presented the lowest invasion rates on all three analyzed axes, as recorded in the TNM notation: L1—14.3% (1/7), V1—14.3% (1/7), Pn1—0% (0/7). No case in this category presented a dissociated pattern (L1V0 or L0V1); the isolated case with vascular invasion presented the concordant positive pattern (L1V1).

Multi-level laryngeal tumors (*n* = 10) presented intermediate rates: L1—40.0% (4/10), V1—20.0% (2/10), Pn1—50.0% (5/10).

Supraglottic (±extension) tumors (*n* = 5) presented high invasion rates on all axes: L1—60.0% (3/5), V1—60.0% (3/5), Pn1—50.0% (2/4).

Hypopharynx (±larynx) tumors (*n* = 26) presented significant rates: L1—34.6% (9/26), V1—34.6% (9/26), Pn1—23.1% (6/26).

**Figure 7:**
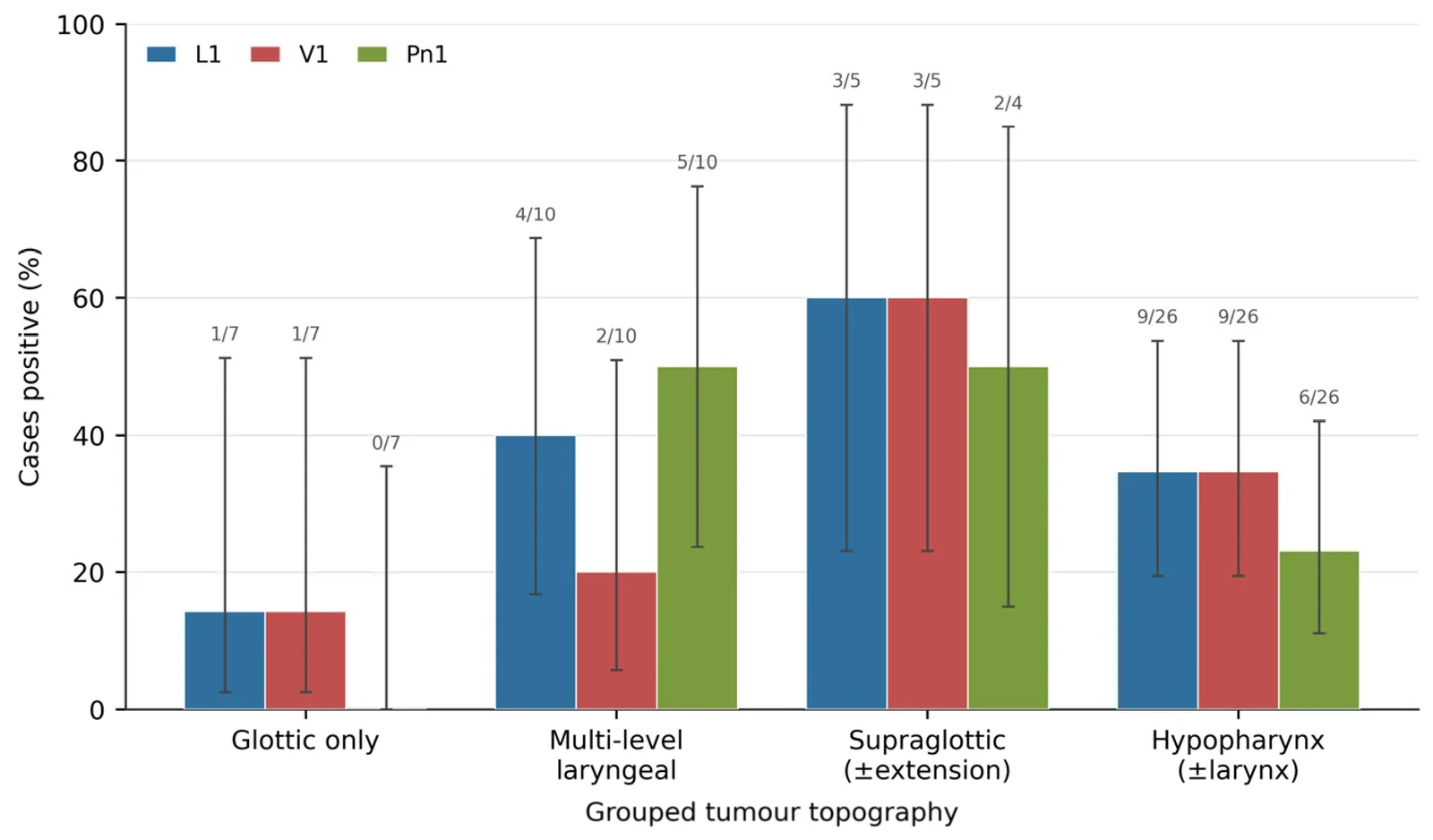
Comparative rates of lymphatic (L1), venous (V1), and perineural (Pn1) invasion across the four grouped tumor topographies. Bars show the proportion of positive cases on each axis within each topographic group (glottic only, *n* = 7; multi-level laryngeal, *n* = 10; supraglottic ± extension, *n* = 5; hypopharynx ± larynx, *n* = 26); error bars are Wilson 95% confidence intervals and absolute case counts (positive/total) are given above each bar. Glottic-only carcinoma presents the lowest values on all three axes (L1 and V1: 14.3%; Pn1: 0%), while supraglottic tumors present the highest. For perineural invasion, the denominator in the supraglottic group is 4 rather than 5, as Pn was not reported in one case. Given the small size of several subgroups, these proportions are descriptive and are not intended to support formal between-group comparisons.

The comparative rates of lymphatic (L1), venous (V1), and perineural (Pn1) invasion across the four grouped topographies are summarized in Figure 7, which illustrates the anatomical gradient of neurovascular-unit involvement: minimal in glottic-only carcinoma, intermediate in multi-level laryngeal tumors, and highest in supraglottic and hypopharyngeal disease.

Fisher’s exact test for the comparison of glottic only vs. the rest of the series revealed consistent trends on all three components, without reaching statistical significance: L1 (OR = 0.26, *p* = 0.40), V1 (OR = 0.32, *p* = 0.41), Pn1 (OR = 0.00—no perineural invasion observed in the glottic-only group, *p* = 0.17). For the overall distribution of perineural invasion across the four grouped topographies, more than half of the expected cell counts fell below five (minimum 1.11), so the chi-square approximation was not applicable; a Fisher–Freeman–Halton exact test was used instead and revealed a statistical trend (*p* = 0.092).

The observed pattern of the anatomical gradient—minimal vascular and perineural invasion at the glottic-only level, intermediate at the level of multi-level laryngeal tumors, and high at the supraglottic and hypopharyngeal levels—is the most consistent descriptive pattern in this series, but it is not established by it. None of the pairwise comparisons reached significance and the Wilson 95% confidence intervals for the glottic-only subgroup are correspondingly wide (L1 and V1, 2.6–51.3%; Pn1, 0–35.4%), so the gradient is reported as hypothesis-generating rather than as a demonstrated topographic effect.

### 3.9. The Neurovascular Unit as an Integrated Axis

To test whether the lymphatic, venous, and perineural axes behave as a single integrated compartment rather than as independent parameters, a composite neurovascular-unit score (NV = L + V + Pn; range 0–3) was analyzed in the 47 cases with reported Pn. The three components showed acceptable internal consistency (KR-20 = 0.725), and the composite score correlated strongly with each of its constituents (NV–L ρ = 0.852; NV–V ρ = 0.812; NV–Pn ρ = 0.688; all *p* < 0.001), supporting their treatment as indicators of a common construct. The distribution of the NV score is summarized in Table 4: half of the cases were fully spared (NV = 0, 51.1%), whereas the neurovascular unit was involved on at least two axes in 27.7% and on all three axes in 14.9%.

Notably, the composite NV score correlated with pT stage more strongly than any single axis analyzed separately (NV–pT: Spearman ρ = 0.534, *p* < 0.001; cf. pT–L1 ρ = 0.365, pT–V1 ρ = 0.390, pT–Pn ρ = 0.422), with a monotonic increase in mean NV score from early to advanced stages (T1/T2 = 0.25 → T3 = 1.31 → T4 = 1.50). This indicates that the unit as a whole tracks tumor stage better than its individual components. All Spearman correlations reported throughout Section 3.5, Section 3.6, Section 3.7, Section 3.8 and Section 3.9 are summarized in Table 5.

The composite score also sharpened the topographic contrast. Glottic-only carcinoma showed the lowest mean NV score (0.29) versus all other topographies combined (1.02), a difference that did not reach conventional two-sided significance (Mann–Whitney U, two-sided *p* = 0.075; one-sided *p* = 0.038 under the a priori directional hypothesis) and is therefore reported as a trend; the four-group Kruskal–Wallis comparison likewise remained non-significant given the small subgroup sizes (H = 3.88, *p* = 0.274). The composite axis thus brings the glottic-protected pattern closer to detectability than any individual L, V, or Pn axis (Section 3.8), without establishing it.

Consistent with an integrated behavior, concordant vascular invasion (L1V1) was strongly associated with perineural involvement: Pn1 was present in 58.3% of L1V1 cases versus 17.2% of L0V0 cases (OR = 6.72, Fisher *p* = 0.020). Because the two vascular components were highly collinear (L–V ρ = 0.722), entering L and V as separate predictors of Pn diluted their individual effects (both non-significant); however, the number of involved vascular axes (0/1/2) predicted perineural invasion in unadjusted analysis (Spearman ρ = 0.349, *p* = 0.016; logistic OR = 2.54 per additional axis, *p* = 0.016). This supports a compartment-level reading of the vasculature rather than two independent channels; as shown in Section 3.7, however, this effect was no longer significant after adjustment for pT stage (adjusted OR = 1.83, 95% CI 0.81–4.16, *p* = 0.148).

## 4. Discussion

The present study analyzes the patterns of vascular invasion in laryngo-hypopharyngeal squamous cell carcinoma from a perspective centered on the differentiated response of vascular compartments to the tumor microenvironment. Unlike conventional approaches that treat lymphovascular invasion (LVI) as a unitary dichotomous parameter, the analysis presented disaggregates this phenomenon into distinct components—lymphatic (L), venous (V), and perineural (Pn)—and examines the relationships between these in relation to tumor topography and T stage. The study group of 48 patients with complete TNM reporting allowed the identification of four key observations which, integrated, provide a conceptual framework for understanding vascular behavior in this type of neoplasia.

### 4.1. Co-Variation in Neurovascular Structures in the Tumor Microenvironment

A consistent co-variation was observed between perineural invasion and both components of vascular invasion (unadjusted Spearman ρ = 0.359, *p* = 0.013 for Pn–L; ρ = 0.325, *p* = 0.026 for Pn–V). This association translates into a distinct clinico-pathological reality: 58.3% of cases with the L1V1 pattern present concomitant perineural invasion, compared with only 17.2% of cases with the L0V0 pattern. As shown in Section 3.7, however, this co-variation was substantially attenuated once tumor stage was accounted for; the interpretation that follows should therefore be read as a hypothesis generated by the descriptive data rather than as a stage-independent effect demonstrated by this cohort.

The previous anatomical study identified the myolaryngovocalis as the layer exhibiting the highest density of closely associated vascular and neural structures. The present observations indicate that these same anatomical relationships are preferentially involved during tumor invasion, consistent with the concept of a neurovascular unit rather than isolated vascular or neural compartments.

The normal histoarchitecture illustrated by the archival Goldner trichrome preparations further supports this interpretation by preserving the spatial organization of the laryngeal neurovascular unit. Unlike immunohistochemical staining, which identifies individual vascular or neural elements, Goldner trichrome retains the architectural context, demonstrating that intramuscular blood vessels and peripheral nerve bundles consistently course together within the connective tissue septa of the myolaryngovocalis muscle. This close anatomical association provides a plausible structural substrate for the coordinated vascular and perineural involvement observed in the present clinicopathological series.

This co-variation pattern is compatible with the hypothesis that the blood vessels, lymphatic vessels, and nerves in the tumor vicinity are affected as a unitary system rather than as isolated structural elements [19,20]. Such a reading would align with the concept of the neurovascular-stromal microenvironment as an integrated functional unit, although the present series cannot establish it independently of tumor stage. Previous immunohistochemical analyses, complemented by the preserved tissue architecture demonstrated in the archival Goldner preparations, indicate that vessels and nerve fibers within the vocal fold are organized as closely associated neurovascular complexes rather than independent anatomical structures [2]. This co-distribution is not merely inferred: in normal human vocal fold tissue, our group recently quantified a parallel, layer-specific spatial distribution of CD31-positive microvascular, NSE-positive neural, and extracellular matrix components, including the biomechanically specialized pericytes of the vocal fold mucosa [21,22] with microvascular density decreasing in a consistent gradient from the vocalis muscle (19.11 ± 6.22 vessels/field) to the superficial (13.55 ± 3.93) and deep (8.11 ± 2.41) lamina propria, and neural profiles following the same topographic pattern [17]. The present study should be interpreted as the pathological continuation of our previous anatomical observations. Whereas our earlier work established the existence of a highly organized neurovascular unit in the normal vocal fold, the present results are consistent with the possibility that this same anatomical unit is engaged as an integrated target during tumor progression. Whether what constitutes a unit of function in normal tissue also constitutes a unit of vulnerability in the tumor microenvironment remains a hypothesis that this cohort supports descriptively but cannot confirm.

In the context of laryngeal carcinoma, the present findings are compatible with the possibility that the anatomical proximity of vascular and neural structures may contribute to partially overlapping patterns of tumor involvement. Potential biological mechanisms may include modifications of the perineural and perivascular extracellular matrix, mediated by metalloproteinases, growth factors such as VEGF, NGF and BDNF, and alterations in pericyte function [23,24,25]. These mechanisms remain hypothetical in the present cohort and were not directly investigated. This interpretation invites a reconsideration of the traditional perspective on perineural invasion, which is usually treated as an event independent of vascular invasion. Our descriptive data is compatible with the possibility that, in laryngo-hypopharyngeal carcinoma, the conceptual separation of the two entities does not fully reflect the biology of the tumor microenvironment. It must be stressed, however, that the present cohort cannot demonstrate that the neural and vascular axes co-vary independently of tumor stage, and that the question therefore remains open.

This integrated behavior can be further explored by modeling the three axes as a single construct. The composite neurovascular-unit score showed acceptable internal consistency (KR-20 = 0.725) and correlated with tumor stage more strongly than any of its individual components (ρ = 0.534 vs. 0.365–0.422). Part of this gain is expected on psychometric grounds, since aggregating correlated binary items reduces measurement error, and the internal consistency of three collinear items reflects that collinearity rather than independently establishing a biological unit. The composite score is therefore proposed as a pragmatic descriptive read-out of neurovascular involvement rather than as proof of an integrated functional compartment. Its ability to accentuate the descriptive contrast between glottic-only and other tumors is hypothesis-generating and requires validation in a larger cohort. Methodologically, the strong collinearity between the lymphatic and venous components (ρ = 0.722) cautions against interpreting L and V as fully independent predictors—a reframing that aligns the statistical structure of the data with the anatomical organization of the neurovascular-stromal unit described in the normal vocal fold [17].

### 4.2. Vascular Invasion Gradient with T Tumor Stage

The second major statistical result confirms the parallel progression of vascular invasion with the advancement of T stage: the overall LVI rate increased from 12.5% in T1 to 57.1% in T3 and T4, with a significant Spearman correlation (ρ = 0.393, *p* = 0.006). This correlation remained consistent for both vascular components analyzed separately (ρ = 0.365 for L1, *p* = 0.011; ρ = 0.390 for V1, *p* = 0.006).

This gradient reflects two complementary biological processes. On the one hand, more advanced tumors have a cumulatively greater probability of contacting and invading vascular structures, by virtue of simple spatial expansion. On the other hand, however, the magnitude of the increase (from 12.5% to 57.1%, a more than fourfold multiplication) suggests that advanced tumors qualitatively change the vascular microenvironment in their vicinity, not just encounter it geometrically more often. T3 and T4 tumors present pro-angiogenic, pro-invasive, and pro-degradative extracellular matrix transcriptional programs significantly more pronounced than T1–T2 tumors, mediated through VEGF-A, MMP-1, MMP-9, and the loss of pericyte–endothelium cooperation [25,26,27,28,29,30]. These modifications, described in detail in our team’s previous review on vocal cord microvascularization [15], become operative in the tumor vicinity and create permissive conditions for the intravasation of tumor cells through compromised vascular walls.

Importantly for clinical interpretation, the LVI rate in T3 (57.1%) is identical to that in T4 (57.1%), suggesting that the key transition for vascular invasion takes place between T2 and T3—that is, at the moment of crossing the anatomical barriers of the larynx (thyroid cartilage, deep paraglottic space, pre-epiglottic structures). This “T2→T3 transition window” may have value for adjuvant therapeutic stratification, but confirmation of this observation requires larger cohorts, with correlation to survival outcomes.

### 4.3. Dissociation Between Lymphatic and Venous Invasion

The third important observation of the study is the existence of a non-negligible proportion of cases with a dissociated vascular invasion pattern as recorded in the TNM notation: 6 of 48 (12.5%) present either L1V0 or L0V1, but not both. The distribution of these dissociated cases is asymmetric, with a 2:1 ratio in favor of isolated lymphatic invasion (4 L1V0 vs. 2 L0V1).

The asymmetry observed in favor of isolated lymphatic invasion is suggestive of a reflection of regional microvascular anatomy. The lymphatic network of the larynx and hypopharynx presents a heterogeneous distribution, with reduced density at the glottic level, but abundant at the supraglottic and hypopharyngeal levels [2,31]. The supraglottic and hypopharyngeal lymphatic vessels are thin-walled structures, lacking circumferential smooth muscle with a barrier role, and with an organization oriented toward drainage to cervical lymph nodes. In contrast, deep venous vessels have thicker walls and a more stable endothelium–pericyte cooperation. This architectural difference provides a plausible explanation for why, when dissociation appears, it tends to manifest more frequently as pure lymphatic invasion.

It must be honestly emphasized that the reduced size of the discordant subgroup (*n* = 6) limits the statistical power of the McNemar test (*p* = 0.683), which could not confirm the observed asymmetry as significant. This observation therefore remains conceptual and descriptive, requiring confirmation in larger cohorts. Stratifying these six discordant cases by topography and stage shows that they cluster in no single anatomical or stage category. Four were L1V0—glottic + subglottic (pT3), transglottic (pT3), and two hypopharyngeal ± laryngeal tumors (pT1 and pT4)—and two were L0V1, both hypopharyngeal ± laryngeal (pT2 and pT4). Both isolated venous cases therefore arose in the hypopharyngeal group, and only one of the six (the L1V0 hypopharyngeal pT1 tumour) showed concurrent perineural invasion; case-level detail is given in Table S2. This distribution is compatible with the anatomical interpretation proposed above but does not support it: with four and two events respectively, no site or stage pattern can be distinguished from chance, and the possibility that part of this discordance reflects morphological misclassification of vessel type (Section 4.6) cannot be excluded. However, the observed pattern is consistent with anatomical predictions and represents a testable hypothesis for further studies.

### 4.4. The Anatomical Gradient of Vascular and Perineural Invasion: The Particular Case of Glottic-Only Carcinoma

The fourth observation integrates the L, V, and Pn data with tumor topography and reflects an anatomical paradigm already described, but now quantified in this case series. Glottic-only carcinoma (limited to the true vocal cord) presented minimum values on all three analyzed axes: L1—14.3%, V1—14.3%, Pn1—0%. In contrast, carcinomas with supraglottic or hypopharyngeal localization presented substantially higher rates on all components.

This difference can be interpreted in light of the vascular features of the glottic region. The true vocal cord is characterized by reduced lymphatic vascularization and a parallel tree-like organization of vessels, with absence of lymphatic drainage at the level of the superficial lamina propria (Reinke’s space) [1,2]. This anatomical particularity, already described in our previous review on vocal cord microvascularization [15], has well-known clinical consequences: the favorable prognosis of limited glottic carcinomas and minimal nodal dissemination. Our data add a nuance to this picture: not only lymphatic invasion, but also venous and perineural invasion are reduced at the glottic-only level. The pattern “glottic only = region protected on all axes” suggests that the glottic anatomical particularity is not limited to the lymphatic system, but extends to the entire neurovascular microenvironment.

The biological mechanism that unifies this observation is probably represented by the reduced network of both vascular and neural structures at the true vocal cord level, especially the superficial lamina propria. This area is characterized by a low density of TH-positive adrenergic fibers [4] and a reduced density of regional neurovascular structures [32]. In this relatively “vasculo-neurally poor” tissue context, the tumor simply has fewer target structures to invade, regardless of its intrinsic invasive biological potential. The opposite ends of the gradient are explained by the same anatomical logic. The glottic level marks a developmental and lymphatic watershed: the supraglottis derives from the buccopharyngeal (branchiogenic) anlage and carries a dense, bilaterally draining lymphatic network, whereas the glottis and subglottis derive from the tracheobronchial anlage and are comparatively lymphatic-poor, with the true vocal cord representing the point of minimal drainage [2,31]. This embryological duality accounts for both the sparse glottic involvement and the high supraglottic rates observed in our series (L1 and V1 each 60.0% at the supraglottic level). The hypopharynx, in turn, possesses an abundant submucosal lymphatic plexus oriented toward early regional dissemination, consistent with the substantial invasion rates seen in the hypopharynx ± larynx group (L1 and V1 each 34.6%). The topographic gradient of neurovascular-unit involvement thus recapitulates the regional architecture of the laryngo-hypopharyngeal lymphovascular supply rather than reflecting a uniform, site-independent tumor property [33,34,35].

It must be acknowledged that the differences observed between topographies did not reach statistical significance in inferential testing. In pairwise comparisons of the glottic-only group against all other topographies, Fisher’s exact test yielded *p* = 0.40 for L1, *p* = 0.41 for V1, and *p* = 0.17 for Pn1. For the overall distribution of perineural invasion across the four grouped topographies, more than half of the expected cell counts fell below five, so the chi-square approximation was not applicable; a Fisher–Freeman–Halton exact test was used instead and yielded *p* = 0.092.

The magnitude of the observed differences is nonetheless substantial—most notably a Pn1 rate of 0% (0/7) in glottic-only carcinoma versus 50% (5/10) in multi-level laryngeal and 50% (2/4) in supraglottic tumors. With subgroups of this size, however, such a contrast is fully compatible with sampling variation, and the present data can neither establish nor exclude a genuine topographic effect. The pattern is therefore reported as a hypothesis consistent with the known regional anatomy of the laryngeal lymphatic and neurovascular networks, and one that a larger cohort would be required to test. Based on the observed proportions, a series comprising approximately 20–25 glottic-only cases—roughly three times the number available here—would be needed to detect a difference in this magnitude with conventional power.

### 4.5. Conceptual and Clinical Implications

Taken together, the present observations support a descriptive framework in which vascular and perineural involvement varies with tumor stage and anatomical site. The data are compatible with the possibility that regional neurovascular architecture contributes to these patterns, but the present cohort does not establish a coordinated biological response of the neurovascular-stromal compartment independently of tumor progression.

The apparent increase in vascular involvement between T2 and T3 is hypothesis-generating and warrants evaluation in larger cohorts with oncological follow-up. The present study was not designed to determine whether detailed L, V and Pn reporting improves treatment selection or adjuvant decision-making.

The layer-specific neurovascular organization previously characterized in the normal vocal fold provides an anatomical rationale for investigating these associations. In the present series, the composite NV score should therefore be regarded as a pragmatic descriptive read-out of combined involvement rather than as evidence of a distinct functional tumor compartment. Prospective studies incorporating lymphatic-specific markers, clinical outcomes and external validation will be required to determine the biological and clinical significance of this framework.

### 4.6. Study Limitations

The present study has several limitations that must be transparently acknowledged. The most consequential of these concerns the validation of the L versus V distinction itself.

First, the study group size (*n* = 48) is moderate for a single-center retrospective study and limits the statistical power of subgroup analyses, especially for the observations regarding dissociated L vs. V patterns (*n* = 6) and glottic-only carcinoma (*n* = 7). Confirmation of these observations requires larger multicenter cohorts.

Second, the inclusion criterion—the presence of TNM reporting with at least an L or V component—generated a study group selected from a larger initial set (247 laryngo-pharyngeal cases in the archive). It is possible that the reports including detailed TNM reporting come predominantly from “educationally interesting” or systematically standardized cases in recent years of the study, introducing a potential reporting bias. Our data do not allow the quantification of this bias.

Third, the study is purely retrospective and does not include follow-up data, so we cannot correlate the observed patterns with clinical outcomes (survival, recurrence, distant metastasis). Nodal stage and margin status, which might have served as partial surrogates, were recorded as pNX in 27 of 48 cases and were not documented at all in 38 of 48 cases, respectively, so neither could be used as an outcome proxy. The patterns described are thus descriptive of the moment of diagnosis, not predictive of clinical evolution.

Fourth, immunohistochemical confirmation with CD31 established the endothelial nature of the invaded spaces and NSE the neural identity of the perineural foci, but CD31 is pan-endothelial and cannot separate lymphatic from blood vascular channels; D2-40 (podoplanin) was not available. The assignment of a given focus to the lymphatic or the venous compartment therefore rests on conventional morphological criteria—wall thickness, presence of erythrocytes, endothelial profile—applied by the reporting pathologist. This has a specific consequence for the present findings: misclassification would not merely add noise but could itself generate apparent dissociation, since a single misassigned focus converts a concordant L1V1 case into a dissociated one. The 12.5% dissociation rate should accordingly be read as an upper bound. Prospective series with D2-40/podoplanin co-staining will be required to establish how much of it reflects genuine compartment-specific involvement.

Fifth, the sex distribution of the study group is strongly imbalanced (95.8% male), limiting the generalization of the conclusions to female patients with laryngeal carcinoma, although this distribution reflects the classical epidemiology of the disease.

Sixth, the co-variation between perineural and vascular invasion did not remain statistically significant after adjustment for pT stage. With 13 perineural events and two predictors (6.5 events per variable), the adjusted analyses are underpowered and their confidence intervals are wide: the point estimates remained in the expected direction (adjusted odds ratio 1.83 per additional involved vascular axis, 95% CI 0.81–4.16) but are compatible with both a moderate stage-independent effect and no effect at all. Whether the neural and vascular axes co-vary independently of tumor stage therefore remains an open question, which a cohort on the order of 150–200 cases with complete Pn reporting would be required to settle.

Despite these limitations, the study offers a detailed characterization of vascular patterns in laryngo-hypopharyngeal carcinoma and opens perspectives for translational research on the neurovascular microenvironment in oncological contexts.

## 5. Conclusions

This retrospective study of 48 cases of laryngo-hypopharyngeal squamous cell carcinoma with complete TNM reporting evidenced four integrated observations regarding the behavior of vessels in tumor contexts. First, perineural and vascular invasion (lymphatic and venous) co-vary in unadjusted analysis, although this co-variation could not be shown to be independent of tumor stage in a series of this size; the observation is therefore consistent with—but does not by itself establish—the concept of the neurovascular-stromal microenvironment as a vulnerable functional unit. Second, the magnitude of vascular invasion increases progressively with the T tumor stage, with an apparent key transition between T2 and T3. Third, 12.5% of cases showed a dissociated L versus V invasion pattern, with four L1V0 and two L0V1 cases. Given the small number of discordant events, the observed asymmetry is descriptive only and may reflect either genuine compartment-specific involvement or morphological misclassification of vessel type. Fourth, glottic-only carcinoma showed the lowest invasion rates on all vascular and perineural axes; with only seven such cases and confidence intervals spanning most of the possible range, this is consistent with—but does not establish—the paradigm of the “protected region” at the level of the true vocal cord.

From a diagnostic standpoint, the concomitant evaluation of the lymphatic (L), venous (V), and perineural (Pn) axes—and their integration into a composite neurovascular-unit read-out—may provide a more complete picture of tumor aggressiveness than the classical reporting of lymphovascular invasion as a single entity, using parameters already available in routine TNM reporting. This composite read-out was evaluated only against pathological stage; no follow-up data were available for the cohort, so whether it adds prognostic information beyond established pathological parameters remains untested and cannot be inferred from these results. These findings support further investigation of blood vessels, lymphatic vessels and neural structures as regionally differentiated components of the tumor microenvironment and provide a basis for larger prospective studies of the neurovascular-stromal framework in laryngo-hypopharyngeal carcinoma.

## Figures and Tables

**Table 1:** Demographic and clinico-pathological characteristics of the study group (*n* = 48).

Variable	*n*	%
Demographic		
Age, mean ± SD (years)	61.3 ± 7.6	—
Age, median (range) (years)	62 (37–78)	—
Male sex	46	95.8
Female sex	2	4.2
Rural residence	27	56.2
Urban residence	21	43.8
Tumor staging (pT)		
pT1	16	33.3
pT2	4	8.3
pT3	14	29.2
pT4	14	29.2
Histological grade (G)		
G1 (well-differentiated)	11	22.9
G2 (moderately differentiated)	25	52.1
G3 (poorly differentiated)	1	2.1
GX (undefined grade)	1	2.1
Not reported	10	20.8
Tumor topography (grouped)		
Glottic (only)	7	14.6
Multi-level laryngeal	10	20.8
Supraglottic (±extension)	5	10.4
Hypopharynx (±larynx)	26	54.2

SD, standard deviation. Percentages are calculated as the proportion of total *n* (48). Histological grade was reported in 38/48 cases (79.2%); the remaining 10 cases (20.8%) did not include explicit mention of the grade in the TNM notation.

**Table 2:** Detailed topographic distribution of primary tumors in the study group (*n* = 48).

Primary Topography	*n*	%
Glottic only (limited to the true vocal cord)	7	14.6
Glottic + subglottic	3	6.2
Supraglottic + glottic	5	10.4
Transglottic (glottic + supraglottic + subglottic)	7	14.6
Hypopharynx + larynx	26	54.2

Primary topography is determined based on the anatomical areas identified in the macroscopic description, microscopic description, and surgical specimen description. The definitions of the categories are presented in section Materials and Methods (2.4). For inferential statistical analysis, topographies were grouped into four main categories (see Methods 2.4): glottic only (*n* = 7), multi-level laryngeal (*n* = 10), supraglottic ± extension (*n* = 5), and hypopharynx ± larynx (*n* = 26).

**Table 3:** Distribution of L + V vascular patterns by grouped tumor topography.

Grouped Topography	L0V0	L1V0	L0V1	L1V1	Total
Glottic (only)	6 (85.7%)	0 (0.0%)	0 (0.0%)	1 (14.3%)	7
Multi-level laryngeal	6 (60.0%)	2 (20.0%)	0 (0.0%)	2 (20.0%)	10
Supraglottic (±extension)	2 (40.0%)	0 (0.0%)	0 (0.0%)	3 (60.0%)	5
Hypopharynx (±larynx)	15 (57.7%)	2 (7.7%)	2 (7.7%)	7 (26.9%)	26
Total	29 (60.4%)	4 (8.3%)	2 (4.2%)	13 (27.1%)	48

Values are presented as *n* (% of topographic total). L0V0 = absence of vascular invasion; L1V1 = concurrent lymphatic and venous invasion (concordant positive); L1V0 = isolated lymphatic invasion (dissociated); L0V1 = isolated venous invasion (dissociated). Dissociated patterns (L1V0 + L0V1) totaled 6 cases (12.5%).

**Table 4:** Distribution of the composite neurovascular-unit score (NV = L + V + Pn) in cases with reported perineural status (*n* = 47).

NV Score	Interpretation	*n*	%
0	Neurovascular unit fully spared	24	51.1
1	Single-axis involvement	10	21.3
2	Two-axis involvement	6	12.8
3	Neurovascular unit fully involved	7	14.9
Total		47	100

NV score = sum of lymphatic (L), venous (V), and perineural (Pn) binary status. One case with an L1V1 pattern lacked reported Pn and was excluded from this analysis.

**Table 5:** Spearman correlations between vascular and perineural invasion parameters and pT tumor stage.

Variable Pair	*n*	Spearman ρ	*p* Value	Significance
pT × LVI (any L1 or V1)	48	0.393	0.006	**
pT × L1	48	0.365	0.011	*
pT × V1	48	0.39	0.006	**
Pn × L1	47	0.359	0.013	*
Pn × V1	47	0.325	0.026	*
Pn × pT	47	0.422	0.003	**
L × V	48	0.722	<0.001	***
NV score × pT	47	0.534	<0.001	***
Vascular axis count (L + V) × Pn	47	0.349	0.016	*

Statistical significance levels: * *p* < 0.05; ** *p* < 0.01; *** *p* < 0.001. LVI = overall vascular invasion (any presence of L1 or V1). Spearman coefficients were calculated for binary/ordinal variables, with two-tailed significance testing.

## Data Availability

The original contributions presented in this study are included in the article/Supplementary Materials. Further inquiries can be directed to the corresponding authors.

## Supplementary Materials

The following supporting information can be downloaded at https://www.mdpi.com/article/10.3390/medsci14050527/s1, Table S1: Rates of lymphatic (L1), venous (V1), and perineural (Pn1) invasion by grouped tumor topography and pT tumor stage, with Wilson 95% confidence intervals; Table S2: Case-level detail of the six cases with dissociated lymphatic/venous invasion (L1V0 or L0V1).

## Abbreviations

The following abbreviations are used in this manuscript:

LVI	Lymphovascular invasion
L	Lymphatic invasion (TNM)
V	Venous invasion (TNM)
Pn	Perineural invasion (TNM)
NV	Neurovascular-unit composite score
SCC	Squamous cell carcinoma
CD31	Platelet endothelial cell adhesion molecule-1 (PECAM-1)
NSE	Neuron-specific enolase
KR-20	Kuder–Richardson formula 20
HE	Hematoxylin–eosin
IHC	Immunohistochemistry
