# Supplementary material for: Beyond Lymphovascular Invasion: Site-Dependent Patterns of Lymphatic, Venous and Perineural Involvement in Laryngo-Hypopharyngeal Carcinoma
Source: Med Sci (Basel). 2026 Aug 28;14(5):527. doi: 10.3390/medsci14050527 (PMC13609669; doi:10.3390/medsci14050527)
Supplement: Supplementary file 1 [file medsci-14-00527-s001.zip › medsci-4476707-supplementary.pdf]

Supplementary Materials

Beyond lymphovascular invasion: site-dependent patterns of lymphatic, venous and perineural involvement in laryngo-hypopharyngeal carcinoma

Table S1. Rates of lymphatic (L1), venous (V1), and perineural (Pn1) invasion by grouped tumour topography and pT tumour stage, with Wilson 95% confidence intervals.

Stratum	n	L1 n (%) [95% CI]	V1 n (%) [95% CI]	Pn1 n/N (%) [95% CI]
By grouped topography				
Glottic (only)	7	1 (14.3) [2.6–51.3]	1 (14.3) [2.6–51.3]	0/7 (0.0) [0.0–35.4]
Multi-level laryngeal	10	4 (40.0) [16.8–68.7]	2 (20.0) [5.7–51.0]	5/10 (50.0) [23.7–76.3]
Supraglottic (±extension)	5	3 (60.0) [23.1–88.2]	3 (60.0) [23.1–88.2]	2/4 (50.0) [15.0–85.0]
Hypopharynx (±larynx)	26	9 (34.6) [19.4–53.8]	9 (34.6) [19.4–53.8]	6/26 (23.1) [11.0–42.1]
By pT stage				
pT1	16	2 (12.5) [3.5–36.0]	1 (6.2) [1.1–28.3]	1/16 (6.2) [1.1–28.3]
pT2	4	0 (0.0) [0.0–49.0]	1 (25.0) [4.6–69.9]	0/4 (0.0) [0.0–49.0]
pT3	14	8 (57.1) [32.6–78.6]	6 (42.9) [21.4–67.4]	5/13 (38.5) [17.7–64.5]
pT4	14	7 (50.0) [26.8–73.2]	7 (50.0) [26.8–73.2]	7/14 (50.0) [26.8–73.2]

Values are absolute counts with percentages and Wilson 95% confidence intervals. For perineural invasion (Pn), N is the number of cases in which Pn was reported in the TNM notation (N = 47 of 48); the single case without reported Pn status belonged to the supraglottic (±extension) group and to the pT3 stratum. Several subgroups are small and the corresponding intervals are correspondingly wide; the proportions are descriptive and are not intended to support formal between-group comparisons.

Table S2. Case-level detail of the six cases with dissociated lymphatic/venous invasion (L1V0 or L0V1).

Case	Pattern	Grouped topography	Detailed primary site	pT	Pn	Grade
1	L1V0	Multi-level laryngeal	Glottic + subglottic	pT3	Pn0	G2
2	L1V0	Multi-level laryngeal	Transglottic	pT3	Pn0	NR
3	L1V0	Hypopharynx (±larynx)	Hypopharynx + larynx	pT1	Pn1	G2
4	L1V0	Hypopharynx (±larynx)	Hypopharynx + larynx	pT4	Pn0	G2
5	L0V1	Hypopharynx (±larynx)	Hypopharynx + larynx	pT2	Pn0	NR
6	L0V1	Hypopharynx (±larynx)	Hypopharynx + larynx	pT4	Pn0	G2

L1V0 = isolated lymphatic invasion; L0V1 = isolated venous invasion, both as recorded in the routine TNM notation. Pn = perineural invasion status; NR = histological grade not reported in the TNM notation. Both L0V1 cases arose in the hypopharyngeal group, and only one of the six cases (case 3) showed concurrent perineural invasion. With four and two events respectively, no site or stage pattern in this subgroup can be distinguished from chance. Because the lymphatic versus venous assignment rests on morphological criteria and was not validated with a lymphatic-specific marker, part of the observed discordance may reflect misclassification of vessel type (see Discussion, Section 4.6).
